# Chronic Lymphocytic Leukemia: Novel Therapeutic Targets Under Investigation

**DOI:** 10.3390/cancers17142298

**Published:** 2025-07-10

**Authors:** Madhavi Nayyar, Ricardo C. B. de Menezes, Sikander Ailawadhi, Ricardo D. Parrondo

**Affiliations:** 1 Division of Hematology/Oncology, Mayo Clinic, Jacksonville, FL 32224, USA; nayyar.madhavi@mayo.edu (M.N.); menezes.ricardo@mayo.edu (R.C.B.d.M.); ailawadhi.sikander@mayo.edu (S.A.); 2 Department of Hematology, Hospital Moinhos de Vento, Porto Alegre 90560-032, Brazil

**Keywords:** chronic lymphocytic leukemia, double-refractory CLL, CAR T-cell therapy, T-cell engagers, novel targets, small molecule inhibitors, BTK degraders

## Abstract

CLL is the most prevalent adult leukemia in Western countries, characterized by the accumulation of monoclonal B lymphocytes. Over the past decade, the therapeutic landscape for CLL has undergone significant transformations, primarily due to the introduction of targeted small molecular therapies like BTK inhibitors and BCL-2 inhibitors, that have improved patient outcomes drastically. Despite significant advances, long-term disease management remains challenging for patients with double-refractory CLL, where responses with subsequent therapies are short-lived. Resistance to these therapies can arise through several mechanisms like kinase-altering BTK mutations, alterations in the BCL-2 pathway, and adaptations within the tumor microenvironment, necessitating the exploration of new therapeutic options. This review provides an in-depth overview of the promising novel treatment approaches under investigation in CLL, focusing on advanced cellular therapies (CAR T-cell therapy), T-cell engagers, new monoclonal antibodies, and various next-generation small molecule inhibitors including BTK degraders, PI3K inhibitors, MALT1 inhibitors, c-MYC inhibitors, CDK9 inhibitors, and agents targeting angiogenesis and DNA damage repair. In this review, we will discuss the novel therapeutic targets and agents as well as ongoing trials, emphasizing the potential of these treatments to overcome resistance and meet the unmet needs of patients, particularly those with double-refractory CLL.

## Simple Summary

Chronic lymphocytic leukemia (CLL) is a hematologic malignancy characterized by the accumulation of clonal, mature B lymphocytes. Although the introduction of Bruton tyrosine kinase (BTK) inhibitors and B-cell lymphoma 2 (BCL-2) inhibitors has dramatically improved patient outcomes, resistance to these therapies remains a significant challenge. This review focuses on the latest advances in CLL therapy, particularly novel therapeutic targets under investigation such as CAR T-cell therapy, T-cell engagers, monoclonal antibodies, antibody–drug conjugates, and novel small molecule inhibitors, including BTK degraders, MALT1 inhibitors, c-MYC inhibitors, and CDK9 inhibitors. This article highlights preclinical and clinical evidence supporting these strategies and discusses the potential impact of these new therapies in addressing the unmet needs of patients who develop resistance to both BTK inhibitors and BCL-2 inhibitors (double-refractory).

## 1. Introduction: Current Treatment Landscape in CLL

CLL, the most common leukemia in adults, with more than 200,000 estimated cases and more than 4000 estimated deaths every year, is a clonal lymphoproliferative disorder characterized by the accumulation of mature but functionally defective B cells in the spleen, bone marrow, lymph nodes, and peripheral blood [1]. CLL treatment has transformed in recent years with the use of BTK inhibitors and BCL-2 inhibitors in both treatment-naïve and relapsed/refractory (R/R) settings. With these treatment advancements, progression-free survival (PFS) and overall survival (OS) increased significantly, and the current estimated 5-year relative survival is 88.5% [1,2,3,4].

BTK inhibitors have become foundational therapies in the management of CLL. These agents irreversibly bind to the C481 residue of BTK, thereby blocking downstream B-cell receptor (BCR) signaling pathways essential for leukemic cell survival and proliferation [2]. BTK inhibitors are broadly categorized into two classes based on their binding mechanism: covalent (irreversible) and non-covalent (reversible). Covalent BTK inhibitors, including ibrutinib, acalabrutinib, and zanubrutinib, bind irreversibly to the C481 residue in the BTK active site. In contrast, non-covalent BTK inhibitors, such as pirtobrutinib, bind reversibly to BTK at alternative sites and retain efficacy even in the presence of C481S mutations. This pharmacodynamic distinction has significant implications for resistance mechanisms and sequencing of therapy in CLL. The first-generation BTK inhibitor ibrutinib has demonstrated superior PFS and OS over chemoimmunotherapy in both treatment-naïve and R/R settings, leading to its approval in multiple lines of therapy [5,6,7]. However, use of BTK inhibitors as monotherapy often necessitates continuous administration, which can lead to the development of resistance and adverse events, including cardiovascular complications like atrial fibrillation and hypertension [2]. Acalabrutinib and zanubrutinib, second-generation BTK inhibitors, were developed to improve selectivity and reduce off-target toxicities associated with ibrutinib [8,9,10]. However, second-generation covalent BTK inhibitors are susceptible to similar resistance mechanisms. Additionally, mutations in PLCγ2, a downstream effector in the BCR pathway, can lead to autonomous signaling independent of BTK, conferring resistance [11]. These mutations are often subclonal at first but can expand under therapeutic pressure.

Venetoclax, a potent and selective BCL-2 inhibitor, induces apoptosis by mimicking BH3-only proteins and displacing pro-apoptotic molecules from BCL-2 [12,13]. It has proven highly effective in combination with anti-CD20 monoclonal antibodies such as obinutuzumab or rituximab, achieving deep remissions and measurable residual disease (MRD) negativity in a fixed-duration regimen for both newly diagnosed and R/R CLL [14,15]. Even with venetoclax, there are emerging mechanisms of intrinsic and acquired resistance. Resistance to venetoclax frequently involves alterations in the apoptotic machinery. One mechanism is the upregulation of alternative anti-apoptotic proteins, such as BCL-XL and MCL-1, which compensate for BCL-2 inhibition [16]. Mutations in BCL2, including the G101V mutation, reduce the binding affinity of venetoclax and confer resistance [17].

The sequencing and combination of these therapies are now at the forefront of clinical decision-making. Nevertheless, patients who progress after both BTK and BCL-2 inhibitors (termed double-refractory) face a particularly poor prognosis and represent a critical unmet need in the field.

## 2. The Unmet Need in Double-Refractory CLL: A Clinical Crossroad

Patients treated with BTK inhibitors/BCL-2 inhibitors with exposure to both these agents (regardless of the reason for discontinuation) are termed “double exposed” and patients who are exposed to both a BTK inhibitors and BCL-2 inhibitors and who are believed to be resistant to both classes of agents are defined as “double refractory” patients [18]. This patient population is a rapidly expanding subset due to the earlier use of these agents in frontline settings and acquired resistance to both drug classes. These patients exhibit median OS of 2.2 years, with limited options for durable disease control [19]. Subsequent therapies in this setting yield varied PFS: PI3K inhibitors provide a median PFS of just 5 months with a 40.9% response rate; chemoimmunotherapy results in median PFS of 3 months with ORR of 31.8%; allogenic stem cell transplantation, although limited by patient eligibility, offers PFS of 11 months with ORR of 76.5%; CAR T-cell therapy provides median PFS of 4 months with 85.7% ORR [20].

Resistance and intolerance to covalent BTK inhibitors and venetoclax emerged as critical clinical challenges. This led to the development of reversible, noncovalent BTK inhibitors, most notably pirtobrutinib, which binds to BTK independently of the C481 site and retains activity against ibrutinib-resistant mutations [21]. Pirtobrutinib has shown promising efficacy in heavily pretreated patients, including those with prior exposure to both BTK inhibitors and BCL-2 inhibitors [22]. Pirtobrutinib (BRUIN trial, NCT03740529) has recently gained FDA approval for use in patients with double refractory CLL with ORR of 73.3% (95% confidence interval [CI], 67.3 to 78.7) and PFS of 19.6 months (95% CI, 16.9 to 22.1) [22]. However, the emergence of non-covalent BTK inhibitors has facilitated the identification of additional resistance mechanisms, particularly in patients experiencing disease progression while on pirtobutinib [23]. Pirtobrutinib retains activity against C481-mutant BTK; however, resistance can still develop through additional mutations outside the kinase domain [24]. These include gatekeeper mutations (e.g., T474) and kinase-dead mutations (e.g., L528W, V416L, A428D, M477I, M437R, and C481F/R), which disrupt drug binding while maintaining BTK’s scaffolding function essential for downstream signaling [25,26].

Additionally, combining BTK inhibitors with BCL-2 inhibitors has emerged as a promising strategy to enhance therapeutic efficacy observed with monotherapy [27,28]. For instance, the BRUIN trial (NCT03740529) evaluated the combination of pirtobrutinib (P) and venetoclax (V), with or without rituximab (R), demonstrating improved PFS rates compared to standard chemoimmunotherapy. The study enrolled 25 patients (PV: *n* = 15; PVR: *n* = 10), of whom 68% had previously received covalent BTKi therapy. Importantly, prior venetoclax treatment was not permitted. At a median follow-up of 27.0 months for PV and 23.3 months for PVR, ORR were high: 93.3% (95% CI, 68.1–99.8) for PV and 100% (95% CI, 69.2–100.0) for PVR. Complete responses were achieved in seven patients receiving PV and 3 receiving PVR. After 12 cycles, undetectable minimal residual disease (uMRD) in peripheral blood (<10^−4^) was achieved in 85.7% (PV) and 90.0% (PVR) of patients. Eighteen-month progression-free survival (PFS) rates were 92.9% (95% CI, 59.1–99.0) for PV and 80.0% (95% CI, 40.9–94.6) for PVR. Treatment was generally well tolerated, with no dose-limiting toxicities observed during the initial safety assessment period. The most common grade ≥ 3 adverse events were neutropenia (52%) and anemia (16%). Adverse events led to dose reduction in three patients and discontinuation in two [29].

Recently, Chimeric Antigen Receptor (CAR) T-cell therapy has emerged as a promising approach for treating double-refractory CLL [30]. CAR T-cells exert their antitumor effects through human leukocyte antigen (HLA) independent tumor cell killing, forming a non-classical immune synapse that facilitates the release of perforin, granzyme, cytokines, and engagement of the FAS axis apoptotic pathway to mediate cytotoxicity [31]. Autologous CD19-directed CAR T-cell therapy lisocabtagene maraleucel (liso-cel) recently gained FDA approval on 14 March 2024 for use in patients with CLL, which has progressed following BTK inhibitors and BCL-2 inhibitors, based on the TRANSCEND-CLL-004 study (Phase I/II) [30]. A single infusion of liso-cel was shown to result in an ORR of 42.9% with CR rates of 18.4% and a median PFS of 11.9 months in patients with relapsed or refractory CLL/SLL including patients who were double-exposed and double-refractory. Ongoing advancements in CAR T-cell design, manufacturing, and patient selection continue to refine its role in CLL. Although CD19-directed CAR T-cell therapy has been transformative in other B-cell malignancies, its efficacy in CLL has been tempered by T-cell dysfunction, antigen loss, and immune suppressive effects of the CLL microenvironments [32]. CLL cells themselves are known to induce T-cell exhaustion, limiting the expansion and persistence of CAR T-cells [33]. Moreover, downregulation or mutation of CD19 on CLL cells can lead to antigen escape, a major cause of relapse after CAR T-cell therapy [34].

To address this issue, strategies such as pre-conditioning with ibrutinib have been employed to enhance CAR T-cell fitness and in vivo expansion. Preclinical and clinical data have shown that ibrutinib modulates the T-cell microenvironment, reduces PD-1 expression, and improves CAR T-cell persistence [35,36]. In the TRANSCEND CLL 004 trial, liso-cel showed ORR of 82%, with MRD-negativity in 75% of evaluable patients. Yet, relapse remains common, especially in patients with high disease burden and prior BTK/BCL-2 inhibitor exposure [30]. Combination approach of liso-cel with ibrutinib yielded a higher overall response rate (ORR) of 86% and a complete response (CR) rate of 45%, with the median PFS of 31.4 months [30]. These findings suggest that integrating BTK inhibitors with CAR T-cell therapy may enhance outcomes, warranting further investigation and future studies [37].

Another cellular therapy for younger patients with high-risk, double-refractory disease is allogenic hematopoietic cell transplant (alloHCT) [38]. While alloHCT offers the potential for durable disease control, with a 2 year PFS of 63% and OS of 81%, it is associated with significant risks, including high rates of toxicity and non-relapse mortality (NRM) of 13% [39]. Complications such as graft versus host disease (GVHD) and opportunistic infections contribute to treatment-related deaths, limiting its broader applicability. Given the emergence of novel targeted therapies, alloHCT is considered an option primarily for patients with aggressive treatment resistant disease [39].

While novel therapeutics such as liso-cel and pirtobrutinib have offered therapeutic options for double-refractory CLL patients, outcomes following CAR T-cell therapy are variable and not feasible for many due to age, comorbidities, and mechanisms of resistance such as antigen escape and limited T-cell persistence, and BTK resistance mutations are a common occurrence with pirtobrutinib. There is, therefore, an urgent need to identify novel therapeutic targets that can overcome existing resistance mechanisms, restore apoptotic sensitivity, and enhance anti-leukemic immune responses. Recent advances in cell therapies, T-cell engagers, monoclonal antibodies, antibody–drug conjugates, and small molecule inhibitors are beginning to address these challenges and offer potential solutions for this difficult-to-treat patient population, as will be discussed in this review.

**Figure 1:**
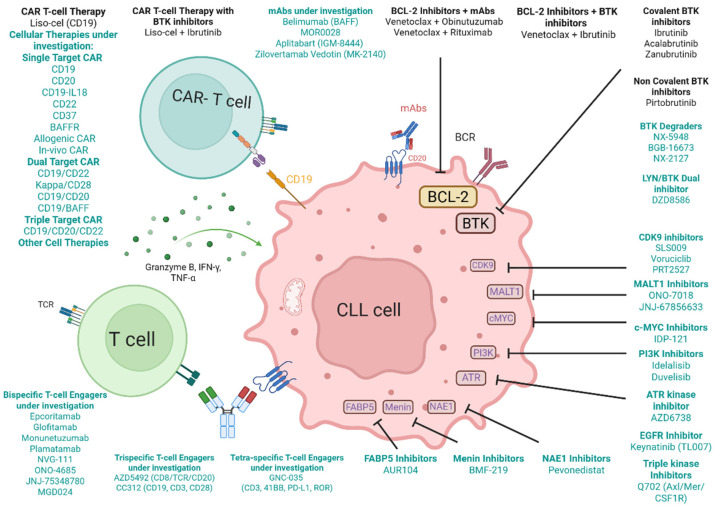
Current treatment landscape and novel therapeutic targets in CLL. Current CLL treatment targets (Black): CAR T cells, CAR T + BTK inhibitors, BCL-2 inhibitors, monoclonal antibodies (mAbs), covalent and non-covalent BTK inhibitors. Novel targets in development (green): Cellular therapies, T-cell engagers, novel monoclonal antibodies, small molecule engagers.

To identify currently active clinical trials in CLL, a systematic literature review was performed in PubMed and across all abstracts from relevant congresses, ASCO, EHA, and ASH, from 1 January 2020 until 1 February 2025 to identify relevant information about novel targets in patients with chronic lymphocytic leukemia, using the search terms of “chronic lymphocytic leukemia“, “double refractory CLL”, “novel targets CLL”, and “clinical trials CLL”. Primary articles that were published in English were assessed for relevancy, to ensure inclusion of all papers and abstracts with clinical data with novel therapies. For clinical trials with multiple data cutoffs, the most recent data were used. The current treatment landscape and emerging therapeutic targets in CLL are illustrated in Figure 1.

## 3. Novel Cellular Therapies in CLL

CAR-T-cell therapy has revolutionized the treatment landscape for various malignancies. While CD19-targeted CAR-T cells have shown efficacy in CLL treatment, relapse remains a significant challenge. Several mechanisms contribute to CAR-T resistance and disease recurrence. One major cause is antigen loss, particularly CD19 downregulation, which allows leukemic cells to evade CAR-T recognition. In some cases, alternative splicing or mutations lead to the expression of CD19 isoforms that are not recognized by the CAR-T construct [40]. In addition to tumor-intrinsic changes, CAR T-cell dysfunction plays a central role in relapse. This includes T-cell exhaustion, characterized by sustained expression of inhibitory receptors such as PD-1, LAG-3, and TIM-3, and a transcriptional profile associated with reduced effector function [40,41]. Over time, CAR T-cells may also exhibit limited persistence, which diminishes long-term immune surveillance and allows minimal residual disease to progress. The tumor microenvironment plays a key role in resistance to CAR T-cell therapy by creating an immunosuppressive milieu. This includes the presence of regulatory T cells, myeloid-derived suppressor cells, and elevated levels of inhibitory cytokines such as TGF-β and IL-10, all of which impair CAR T-cell activation and cytotoxic function. Moreover, upregulation of immune checkpoint pathways, particularly PD-1/PD-L1, can directly suppress CAR T-cell cytotoxicity. These multifaceted resistance mechanisms have prompted the development of next-generation strategies, such as multi-target CARs. Targeting multiple antigens simultaneously may reduce the likelihood of tumor evasion and improve therapeutic outcomes [42].

In addition to commercially developed CAR T-cell products, several academic institutions have played a pioneering role in developing and evaluating CD19-directed CAR T-cell therapies in CLL. For instance, investigators at the University of Pennsylvania conducted one of the earliest studies using autologous CART19 cells in patients with heavily pretreated CLL [43,44]. These academic efforts have contributed significantly to optimizing CAR design, lymphodepletion protocols, and response biomarkers in CLL. Although academic CAR T programs may face scalability and regulatory challenges compared to industry-sponsored trials, they remain a critical engine of innovation and continue to inform commercial development pipelines. Their role is especially relevant in early phase studies exploring next-generation CAR constructs, combinatorial strategies, and mechanisms of resistance in R/R CLL.

Various single, dual, and triple-targeted CAR-T strategies, as well as other cellular therapies currently in clinical trials, are enumerated in Table 1, highlighting their potential in CLL treatment.

### 3.1. Single-Target CAR T-Cell Therapy

CD20-targeting CARs provide another therapeutic option, especially in antigen-escape scenarios [45,46,47]. MB-106 is a third-generation, fully human CD20-targeted CAR-T cell therapy showing an excellent safety profile in NHL, with CRS in only 2 of 16 patients, no ICANS, and high efficacy, including an ORR of 85% and CR of 57% in follicular lymphoma patients, manufactured using an optimized process designed to enhance T-cell expansion and product consistency [48]. Enrollment is ongoing (NCT03277729) with expanded eligibility to all CD20+ CLL with the new amendment.

huCART19-IL18 is a next-generation construct that combines CD19 specificity with IL-18 secretion to improve T-cell expansion and antitumor activity. Recent phase I results from the ongoing trial NCT04684563 highlight the therapeutic promise of huCART19-IL18. In heavily pretreated patients with R/R NHL, including those with prior CD19 CAR T-cell exposure, the therapy demonstrated a favorable safety profile and encouraging efficacy, with a complete or partial response seen in 81% of the patients (90% confidence interval [CI], 62 to 93), complete response in 52% (90% CI, 33 to 71), and partial response in 29% (90% CI, 13 to 49), at 3 months after infusion. With a median follow-up of 17.5 months (range, 3 to 34), the median duration of response was 9.6 months (90% CI, 5.5 to not reached) [49,50]. The construct’s ability to induce durable remissions even after prior CAR T-cell failure suggests that there is retained CD19 targetability and potential benefits of IL-18-mediated immune modulation.

CAR-37 T cells, which target receptor tyrosine kinase-like orphan receptor 1 (ROR1), aim to selectively eliminate malignant B cells while sparing normal tissues [51]. ROR1 is an oncofetal protein that is aberrantly expressed on the surface of CLL cells but largely absent in normal adult tissues, making it an attractive target for immunotherapy. Its restricted expression profile and role in CLL cell survival and proliferation support the development of ROR1-targeted therapies such as CAR-37 T cells [52,53]. CAR-37 T cells have shown promise in preclinical studies and early phase clinical trials for treating CD37+ hematologic malignancies including CLL [54].

B-cell activating factor (BAFF) receptor-targeting CAR T cells, targeting the B-cell activating factor receptor, present another promising strategy due to its restricted expression in malignant B cells. BAFF-R is a member of the tumor necrosis factor receptor family that is essential for B-cell development and survival and is consistently expressed on CLL cells [55]. Its selective expression on malignant B-cells, including those lacking CD19, makes BAFF-R an appealing alternative target for CAR T-cell therapy in R/R CLL. Anti-BAFF-R CAR-T cells demonstrated potent cytotoxicity against CLL cell lines and primary patient-derived tumor cells. Notably, MC10029 CAR T cells were effective even against CD19-negative CLL cells, which are resistant to traditional CD19-targeted therapies [56].

Allogeneic approaches are also gaining traction, such as allogenic CARCIK-CD19 and anti-CD19 allogeneic CAR therapies, which use donor-derived or engineered cells to overcome autologous T-cell limitations [57]. Autologous CAR-T cell treatments rely on collecting and engineering a patient’s own T-cells, a process that is both time-consuming and logistically complex. Manufacturing typically requires several weeks, during which patients with aggressive disease may experience rapid clinical deterioration. Furthermore, the quality of autologous T cells is often compromised in heavily pretreated cancer patients, who may have exhausted, senescent, or functionally impaired T cells. This can result in poor expansion, reduced toxicity, and variability in therapeutic efficacy. Also, the individualized nature of autologous therapies limits scalability and contributes to high production costs and occasional manufacturing failures when insufficient or poor-quality cells are obtained. CARCIK-CD19 is an investigational allogeneic CAR-T cell therapy engineered using the non-viral Sleeping Beauty transposon system. It targets CD19 and employs cytokine-induced killer (CIK) cells derived from haploidentical donors, which possess both T cell and natural killer (NK) cell cytotoxic properties. By using healthy donor-derived cells, CARCIK-CD19 offers a scalable, off-the-shelf approach that can overcome the production delays, variability, and reduced cell fitness often encountered with autologous CAR-T cell therapies [58].

Additionally, UB-VV111 represents an innovative platform that generates CD19 CAR T cells in vivo, streamlining the manufacturing process and reducing time to treatment. UB-VV111 utilizes the VivoVec™ gene delivery platform, which combines a third-generation lentiviral vector with a novel T-cell targeting and activation surface complex [59]. Upon administration, UB-VV111 selectively binds to and transduces T cells in vivo, encoding a transgene for an anti-CD19 CAR and a Rapamycin Activated Cytokine Receptor (RACR™) [60]. The RACR™ system promotes the expansion and persistence of the engineered CAR-T cells within the body. Preclinical studies have demonstrated that a single dose of UB-VV111 can generate CAR-T cells in vivo without the need for lymphodepleting conditioning. In non-human primates, this approach resulted in durable B-cell aplasia for up to 76 days, exceeding industry benchmarks for ex vivo CAR-T therapies [61]. Furthermore, the therapy showed efficient CAR-T cell generation, expansion in response to target antigens, and the formation of memory CAR-T cell populations. It is currently in a Phase I trial (NCT06528301) for patients with R/R large B-cell lymphoma (LBCL) and CLL.

### 3.2. Dual-Target CAR T-Cell Therapy

Dual-targeting strategies have emerged as a powerful approach to reduce antigen escape and enhance treatment efficacy. One promising example is the CD19/CD22 bicistronic CAR, which co-expresses receptors for both antigens to maintain sustained targeting of malignant B cells, especially in cases where one antigen may be downregulated [62]. Similarly, multiple CD19/CD22 CAR constructs, whether designed as tandem or co-expressed receptors, have demonstrated potential in preclinical and early phase clinical studies to enhance antigen coverage and reduce relapse due to single-antigen loss [63]. Another dual-targeted construct, the CD20/CD19 CAR, is currently under investigation in CLL [64]. Additionally, the CD19-BAFF CAR represents an innovative strategy that combines CD19 specificity with a BAFF-binding domain, enabling recognition of malignant cells expressing either target and improving cytotoxic efficacy in tumors with heterogeneous antigen expression [65]. Kappa-CD28 CAR T cells represent a subtype-specific strategy, designed to selectively target kappa light chain-expressing malignant B cells, while sparing healthy lambda-expressing B cells, which may help preserve some normal immune function [66]. Together, these dual-targeted CAR approaches represent an evolving frontier in CLL therapy, designed to overcome the limitations of single-antigen targeting and deliver more robust, long-lasting responses.

### 3.3. Triple-Target CAR T-Cell Therapy

One notable advancement is the development of CAR T cells engineered to simultaneously target CD19, CD20, and CD22 (NCT05418088) [67]. By engaging three distinct surface markers, these CAR T cells can recognize and eliminate heterogeneous tumor populations more effectively, offering enhanced tumor clearance and potentially more durable remissions. Additionally, this multi-target strategy helps maintain anti-tumor efficacy even if one or two of the target antigens are lost or downregulated during disease progression. As research advances, the CD19/CD20/CD22 triple CAR represents a promising next step in optimizing CAR T-cell therapy for CLL, with the potential to achieve deeper and more lasting clinical responses.

### 3.4. Other Cellular Therapies in Clinical Trials

Beyond traditional CAR T-cell therapy, a range of innovative cellular therapies are being explored for the treatment of CLL, offering new avenues to enhance efficacy, safety, and accessibility. Autologous peripheral blood lymphocytes (NCT04155710) harvested from CLL patients previously treated with ibrutinib have potential immune-stimulating and antineoplastic activities [68,69]. Kappa-CD28 T-cells (NCT00881920), which selectively target kappa light chain-expressing B cells, offer a subtype-specific approach that spares healthy lambda B cells and preserves some immune function [70]. Another promising strategy involves the use of autologous or syngeneic peripheral blood T lymphocytes and Epstein–Barr virus-specific cytotoxic T lymphocytes (EBV-CTLs) engineered to express CD19 CARs (NCT00709033), combining tumor specificity with the antiviral activity of EBV-CTLs to enhance persistence and efficacy [71]. Allogeneic NK T-cell based therapies are also gaining traction, such as KUR-502 (NCT05487651), which utilizes CD19-specific CAR-expressing NK T-cells derived from healthy donors [72]. Combinatorial strategies are also being explored, such as pairing NK cells with IL-2 and a TGFβ receptor 1 inhibitor (NCT05400122) to enhance NK cell activation and overcome the immunosuppressive tumor microenvironment [73]. Together, these diverse cellular therapies may overcome current limitations and offer more flexible, durable, and widely available immunotherapeutic options.

## 4. T-Cell Engagers in CLL

T-cell engagers represent a novel and highly promising class of immunotherapies in CLL, designed to redirect polyclonal cytotoxic T lymphocytes toward malignant B cells via synthetic linkage. Unlike the current CAR T-cell therapies for CLL, which require ex vivo manipulation, T-cell engagers are off-the-shelf, antibody-based constructs with the capacity to overcome T-cell exhaustion and antigen escape. Their modular structure allows for targeting of multiple antigens and engagement of co-stimulatory pathways, making them a versatile platform for drug development in double-refractory CLL. Several active clinical trials evaluating T-cell engagers in CLL in their early study phases are enumerated in Table 2.

### 4.1. Bispecific T-Cell Engagers

Several novel agents are in development or early clinical evaluation. Epcoritamab, a subcutaneously administered CD3 × CD20 Bispecific T-cell engager (BiTE), received commercial approval in May 2023 for the treatment of R/R diffuse large B-cell lymphoma [74]. Preliminary findings from phase I/II EPCORE 1-CLL (NCT04623541) have shown encouraging efficacy in patients with high-risk disease and those previously exposed to multiple lines of therapy. Epcoritamab was administered in a step-up dosing schedule, starting with weekly doses in cycles 1–3, biweekly in cycles 4–9, and monthly in cycles ≥ 10. In the dose-escalation phase, seven patients received epcoritamab at 24 mg (3 patients) and 48 mg (4 patients) [75]. No dose-limiting toxicities occurred, and common side effects included cytokine release syndrome, fatigue, injection-site reactions, and nausea. CRS occurred in all patients but was limited to grade 2. There were no cases of immune effector cell-associated neurotoxicity syndrome (ICANS) or tumor lysis syndrome. Antileukemic activity was observed at both dose levels, with partial responses in three out of five patients who completed the full response assessment. This study will present updated data on clinical outcomes and pharmacokinetics for additional patients.

In addition to epcoritamab, other CD20 × CD3 BitEs, such as glofitamab (NCT03075696) and mosunetuzumab (NCT02500407), are approved for various B cell malignancies. FDA granted approval to mosunetuzumab-axgb for R/R follicular lymphoma in December 2022, and to glofitamab-gxbm for selected R/R large B-cell lymphomas in June 2023 [76,77]. However, to date, no CLL-specific results have been published with these agents.

Various other BiTEs targeting molecules CD3 and CD20 are currently under investigation for CLL, including plamotamab (NCT02924402) and GB261 (NCT04923048) [78,79]. BiTEs targeting molecules other than CD3 and CD20 are also under active trials for CLL. For instance, NVG-111 (NCT04763083) is a novel bispecific that targets ROR1 and CD3, harnessing T cells against a more selective target to reduce off-tumor toxicity [80]. Additionally, ONO-4685 (PD-1 × CD3) (NCT06547528) represents a unique approach by engaging T cells while potentially modulating immune checkpoint pathways, possibly reinvigorating exhausted T cells within the tumor microenvironment. JNJ-75348780 (NCT04540796), a CD3 × CD22 BiTE, is being explored as a strategy to overcome resistance in CD19-low or -negative disease [81]. Another agent, MGD024 (NCT05362773), is a CD123 × CD3 BiTE currently in dose escalation trials [81]. Collectively, these bispecific engagers reflect a highly dynamic and promising area in CLL therapy, providing flexible immunotherapy options with the potential for deep and durable responses, especially in patients not eligible for or relapsing after CAR T-cell therapy.

### 4.2. Trispecific Antibodies

Trispecific T-cell engagers are antibody-based constructs engineered to simultaneously bind three distinct targets, most commonly involving CD3 on T cells and two tumor-associated antigens on malignant cells [82]. For instance, trispecific antibodies designed to target CD3 on T cells and both CD20 and CD22 on malignant B cells facilitate the redirection of cytotoxic T cells toward tumor cells expressing either or both surface antigens. This dual-antigen targeting approach enhances therapeutic coverage and may reduce the risk of antigen escape compared to monospecific or bispecific formats [83].

Another major design class of trispecific engagers incorporates a co-stimulatory receptor, such as CD28 or 4-1BB, alongside CD3 and a single tumor-associated antigen, to enhance T-cell activation, proliferation, and persistence. One example is AZD5492, part of the TITANium study (NCT06542250), which utilizes a CD8/TCR-based T-cell engaging antibody targeting CD20 and is currently being evaluated in a Phase I/II trial for R/R B-cell malignancies, with study completion expected in 2028 (NCT06542250). Another trispecific, CC-312 (NCT06037018), targets CD19, CD3, and CD28, aiming to not only recruit T cells via CD3 but also deliver a potent co-stimulatory signal through CD28, improving the expansion and activity of T cells in CD19-positive CLL.

### 4.3. Tetraspecific Antibodies

Tetraspecific T-cell engagers are engineered molecules designed to simultaneously bind four distinct antigens or receptors, enabling the targeting of multiple signaling pathways or immune cell engagement to enhance therapeutic efficacy [84,85], such as GNC-035 (NCT05944978), which are under investigation in CLL and other hematological malignancies. GNC-035 targets CD3, 4-1BB, PD-L1, and ROR1 providing T-cell activation (CD3), co-stimulation (4-1BB), checkpoint inhibition (PD-L1), and tumor specificity (ROR1) within a single molecule [86]. These novel multi-specific engagers offer a powerful and highly tailored immunotherapeutic strategy.

## 5. Monoclonal Antibodies and Antibody–Drug Conjugates in CLL

Novel monoclonal antibodies are playing an increasingly important role in the evolving therapeutic landscape of CLL, offering targeted mechanisms that may overcome resistance to current therapies. One such agent is belimumab, an anti-BAFF antibody currently being evaluated in the BeliVeR trial (NCT05069051). BAFF (B-cell activating factor), a member of the TNF family, supports malignant B-cell survival and has been implicated in resistance to agents such as ibrutinib and venetoclax by inhibiting apoptosis and maintaining mitochondrial integrity [87]. A study by Tandler et al. demonstrated that BAFF neutralization with belimumab enhanced the sensitivity of CLL cells to idelalisib, ibrutinib, and venetoclax, irrespective of clinical stage (Binet or Rai) or IgHV mutational status. Given belimumab’s prior approval in autoimmune diseases, its repurposing as an adjunct to targeted therapy in CLL represents a promising therapeutic strategy currently under clinical investigation [87].

MOR00208 (NCT02005289) is a new anti-CD19 monoclonal antibody designed to target CD19-positive B cells, potentially enhancing outcomes when used in combination with other agents. MOR00208 is engineered with modifications to its Fc region, specifically, the S239D and I332E mutations, which enhance its ability to activate immune responses. These modifications increase antibody-dependent cell-mediated cytotoxicity (ADCC) and phagocytosis, making it more effective at targeting and eliminating cancerous B cells compared to unmodified antibodies [88].

CAP-100 (NCT04704323), a humanized antibody targeting C-C chemokine receptor 7 (CCR7), aims to interfere with CLL cell trafficking and homing to protective niches like lymph nodes, potentially sensitizing the cells to therapeutic attack [89]. Aplitabart (IGM-8444) (NCT04553692), an IgM DR5 agonist antibody, leverages the apoptotic pathway by binding to death receptor 5 (DR5) to induce programmed cell death in CLL cells, independent of the p53 status, which is crucial in high-risk cases [90]. Meanwhile, zilovertamab vedotin (MK-2140), being evaluated in the waveLINE-006 study (NCT05458297), is an antibody–drug conjugate targeting ROR1 linked to monomethyl auristatin E (MMAE), a cytotoxic agent [91]. This allows for precise delivery of the payload directly to malignant cells, minimizing off-target effects. Preliminary results of this ongoing waveLINE-001 study with outcomes for patients with mantle cell lymphoma (MCL), diffuse large B-cell lymphoma (DLBCL), and Richter transformation (RT) have been published [92].

Collectively, these novel monoclonal antibodies offer diverse and complementary mechanisms that may significantly enhance the depth and durability of responses in CLL, particularly in double-refractory patients. Active clinical trials of monoclonal antibodies and antibody–drug conjugates in CLL are enumerated in Table 3.

## 6. Small Molecule Inhibitors in CLL

The development of novel small molecule inhibitors has significantly expanded the therapeutic options for patients with relapsed, refractory, or high-risk CLL. Novel small molecule inhibitors far advanced in clinical development in CLL are enumerated in Table 4.

Among these, second-generation BCL-2 inhibitors, including sonrotoclax (BGB-11417), lisaftoclax (APG-2575), ABBV-453, and mesutoclax (ICP-248), demonstrate enhanced potency and selectivity over venetoclax, with the potential to overcome resistance mechanisms and reduce toxicity [93]. In parallel, second-generation covalent BTK inhibitors, such as zanubrutinib, tirabrutinib, and orelabrutinib, have been designed to improve kinase selectivity and reduce off-target effects associated with earlier BTK inhibitors like ibrutinib. These agents show promise in improving tolerability while maintaining clinical efficacy [94].

Sonrotoclax (BGB-11417), a next-generation BCL-2 inhibitor, has shown deep and durable responses in combination with zanubrutinib. In the BGB-11417-101 study (NCT04277637), the combination achieved a 97% ORR and 57% CR, with 100% ORR and 73% CR at the 320 mg dose. Undetectable MRD in blood was seen in 85% of evaluable patients, with no tumor lysis syndrome or dose-limiting toxicities observed [93,94].

Lisaftoclax permits rapid daily ramp-up and has demonstrated an ORR of 96.6% in combination with acalabrutinib. In patients with prior venetoclax exposure, responses were preserved, with ORRs of 85.7%, including 100% in BTKi-naïve and 66.7% in double-exposed patients. No treatment-related discontinuations were attributed to lisaftoclax [95].

Non-covalent BTK inhibitors, such as nemtabrutinib (MK-1026), vecabrutinib, fenebrutinib, CGI-1746, GDC-0834, and RN486, are specifically designed to retain activity against BTK mutations (e.g., C481S) that confer resistance to first-generation covalent BTK inhibitors [103]. In the BELLWAVE-001 study (NCT03162536), nemtabrutinib demonstrated an ORR of 56% with a median PFS of 26.3 months among R/R CLL patients, and an ORR of 58% with median PFS of 10.1 months in the double-exposed subgroup [96].

LP-168 represents a novel dual covalent and non-covalent BTK inhibitor (fourth generation), aiming to combine the benefits of both binding mechanisms for broader efficacy [98]. However, resistance to noncovalent BTK inhibitors, such as pirtobrutinib, can also arise through on-target mutations in the BTK kinase domain, including V416L, A428D, M437R, T474I, and L528W, which may interfere with inhibitor binding while maintaining kinase activity. These mutations are structurally distinct from C481S and cluster around critical residues involved in reversible inhibitor interactions [104]. In addition to these BTK mutations, several studies have identified alternative mechanisms of resistance involving upregulation of parallel survival pathways such as PI3K/AKT, MAPK/ERK, and NF-κB signaling, which can bypass BTK inhibition and maintain leukemic cell viability. These compensatory pathways are especially relevant in patients who progress without identifiable BTK or PLCγ2 mutations, suggesting a broader resistance phenotype driven by pathway redundancy and microenvironmental interactions.

While BTK and BCL-2 inhibitors remain central to CLL therapy, there is growing interest in alternative small molecule targets that may overcome resistance and improve outcomes. This section explores emerging pathways and mechanisms relevant to CLL pathogenesis and resistance, focusing on small molecule targets beyond the well-established classes of covalent/non-covalent BTK inhibitors and BCL-2 inhibitors.

An emerging class, BTK degraders, including NX-5948, BGB-16673, NX-2127, NRX-0492, ABBV-101, HZ-Q1060, and AC676, use targeted protein degradation to eliminate the BTK protein entirely, offering an innovative approach to bypass kinase domain mutations. BTK degraders utilize proteolysis-targeting chimeras (PROTACs) or molecular glues to induce ubiquitin-proteasome-mediated degradation of both wild-type and mutant BTK, regardless of binding site conformation [105]. By degrading the entire BTK protein, these agents may retain efficacy in the presence of both covalent and noncovalent BTK inhibitor- resistant BTK mutations, including those located outside the C481 residue. These agents offer the possibility of rescuing double-refractory patients with a non-kinase, degradation-based strategy which affects BTK’s scaffolding function.

Additionally, by eliminating the BTK protein altogether, degraders may counteract resistance mechanisms associated with enhanced BTK adaptor functions or conformational changes that maintain downstream BCR signaling independent of catalytic activity. This approach may also be beneficial in cases wherein leukemic cells rely on BTK-mediated scaffolding to assemble multi-protein signaling complexes that cannot be disrupted by traditional inhibitors.

Preliminary data from the phase I NX-5948-301 study for NX-5948 demonstrated an ORR of 76.7% in patients with R/R CLL characterized by high-risk features, including central nervous system involvement, prior exposure to BTK and BCL2 inhibitors including pirtobrutinib (97.1% of patients had prior BTK inhibitor therapy, 91.2% had prior BCL2 inhibitor therapy, and 23.5% had prior exposure to pirtobrutinib), TP53 mutations (40.4%), and BTK mutations (38.6%) [100]. The most frequently observed treatment-emergent adverse events (TEAEs) included purpura/contusion (44.1%, with no grade ≥ 3 events), thrombocytopenia (23.5%, including 2.9% grade ≥ 3 events), petechiae (29.4%, with no grade ≥ 3 events), fatigue (20.6%, with no grade ≥ 3 events), and neutropenia (17.6%, including 14.7% grade ≥ 3 events); patients with up to grade 4 cytopenias were eligible for study participation, rash (23.5%, with no grade ≥ 3 events; 2.9% classified as serious adverse events [SAEs]), and headache (23.5%, with no grade ≥ 3 events). Notably, no cases of new onset atrial fibrillation/flutter or hypertension were reported [100].

Similarly, an ongoing phase I study (CaDAnCe-101) is evaluating BGB-16673 in patients with B-cell malignancies. Updated results presented at the 2024 American Society of Hematology (ASH) Annual Meeting demonstrated an ORR of 77.6% among 49 response-evaluable patients with relapsed or refractory CLL, including a complete response in 4.1% of patients [101,106]. TEAEs were observed in 88.5% of patients, with grade ≥ 3 events occurring in 46.2% and serious adverse events in 38.5% of patients. The most commonly reported TEAEs included contusion (30.8%, with no grade ≥ 3 events), pyrexia (23.1%, with no grade ≥ 3 events), neutropenia, or decreased neutrophil count (23.1%, including 15.4% grade ≥ 3 events), and increased lipase levels (23.1%, including 3.8% grade ≥ 3 events; all cases were transient and asymptomatic); importantly, no instances of hypertension or atrial fibrillation were reported [101].

NX-2127 is an orally administered, dual-function small molecule degrader that simultaneously targets BTK for degradation while exerting immunomodulatory effects through the cereblon-mediated degradation of the transcription factors Ikaros and Aiolos [26]. A phase I clinical trial (NX-2127-001; NCT04830137) is currently underway in patients with B cell malignancies. The most recent reported data, as of November 2023, included a median follow-up duration of 9.5 months, though no ORR was provided. The most frequently observed grade ≥ 3 TEAEs were neutropenia (38.3%), hypertension (14.9%), and anemia (12.8%). Atrial fibrillation was reported in 12.8% of patients, with 6.4% experiencing grade ≥ 3 events. The most common reasons for treatment discontinuation were progressive disease (25.5%) and adverse events (21.3%).

Additional small molecular strategies include phosphatidylinositol-3-kinase (PI3K) inhibitors like idelalisib, which inhibit critical PI3K/AKT/mammalian target of rapamycin (mTOR) signaling pathways for CLL cell survival [107,108]. PI3K isoform inhibition in CLL not only exerts direct anti-tumor effects but may also mediate antineoplastic activity through disruption of the CLL microenvironment [109,110,111]. Inhibiting these downstream or parallel survival pathways has therefore emerged as a rational strategy to restore treatment sensitivity. However, their clinical utility has been substantially limited by a narrow therapeutic window and a high incidence of immune-mediated toxicities, including severe colitis, pneumonitis, and transaminitis, as well as an increased risk of opportunistic infections [112]. High rates of treatment discontinuation due to adverse events have further hindered their adoption in clinical practice. These safety concerns have prompted regulatory scrutiny, including black box warnings and, in some cases, market withdrawal (e.g., umbralisib). Despite these limitations, PI3K inhibitors may retain clinical relevance in selecting high-risk settings. In a retrospective analysis by Thompson et al., responses were observed in double-refractory CLL patients with ORR of 46% and median PFS of 5 months. Although response duration was relatively short and toxicity remained a challenge, these findings suggest a potential role for PI3K inhibitors as a bridging or salvage option in heavily pretreated populations. Nevertheless, their clinical impact remains modest compared to emerging therapies such as BTK degraders, which offer improved tolerability and more durable responses. As a result, PI3K inhibitors are typically reserved for later lines of therapy in this high-risk population, and their role is increasingly limited due to concerns over safety and the availability of more effective and better-tolerated therapies [113].

Beyond these, a diverse array of targeted agents is under early investigation, listed in Table 5.

ACY-1215, also known as ricolinostat, is a selective histone deacetylase 6 (HDAC6) inhibitor currently under investigation for the treatment of R/R CLL. Unlike pan-HDAC inhibitors, ricolinostat targets HDAC6 specifically, which plays a key role in protein degradation, cell motility, and immune modulation, potentially offering a more favorable safety profile [114]. It is being evaluated in a Phase I clinical trial (NCT02787369) in combination with ibrutinib or idelalisib. This study reflects a growing interest in epigenetic modulation as a complementary strategy to existing targeted therapies in CLL.

Ataxia telangiectasia and Rad3-related (ATR) kinase is a key regulator of the DNA damage response pathway [115]. Berzosertib, an ATR inhibitor, has shown synthetic lethality in TP53-mutant cells, especially when combined with PARP inhibitors or DNA-damaging agents [116]. Early phase studies are ongoing, and trials exploring ATR inhibition in R/R CLL are planned. For instance, AZD6738 (ceralasertib) is a selective ATR kinase inhibitor being investigated for the treatment of R/R high-risk CLL. ATR plays a crucial role in the DNA damage response, particularly in cells under replicative stress conditions commonly found in high-risk CLL, especially with TP53 mutations or deletions [115]. Ceralasertib is designed to exploit this vulnerability by disrupting DNA repair mechanisms, thereby inducing synthetic lethality in cancer cells. It is currently being evaluated in a Phase I clinical trial (NCT03328273) in combination with acalabrutinib to assess safety, tolerability, and preliminary efficacy.

DZD8586 (TAI-SHAN8) is a dual LYN/BTK inhibitor with blood–brain barrier (BBB) penetration properties. LYN and BTK are both key components of the B-cell receptor (BCR) signaling pathway [117]. Dual inhibition of these kinases may provide more comprehensive pathway blockade and help overcome resistance to BTK inhibitor monotherapy, particularly in high-risk or heavily pretreated patients. Its BBB penetration properties open potential therapeutic avenues for patients with central nervous system involvement, a rare but challenging manifestation in CLL [118]. Updated Phase 2 data (TAI-SHAN9) was presented at the American Society of Clinical Oncology’s (ASCO) 2025 Annual Meeting held in May 2025 [119]. As of 8 January 2025, 39 patients with R/R DLBCL were treated with DZD8586 at 25–75 mg daily. Among 25 efficacy-evaluable patients, ORR were 42.9% at 50 mg and 50% at 75 mg, with CRR of 28.6% and 50%, respectively. Responses were seen in both GCB and non-GCB subtypes. The longest PFS observed was 5.6 months (ongoing). DZD8586 was well tolerated, with mostly mild-to-moderate TEAEs and no reports of atrial fibrillation, bleeding, or treatment-related deaths.

Mucosa-associated lymphoid tissue lymphoma translocation protein 1 (MALT1) is a critical mediator of CBM (CARD11-BCL10-MALT1) complex-driven NF-κB signaling, which is constitutively activated in CLL and contributes to BCR pathway-independent survival [120]. The CARD11-BCL10-MALT1 signaling complex is a pivotal regulator of NF-κB activation in CLL, and its dysregulation has been implicated in promoting resistance to therapies targeting BCR signaling. CARD11, a key adaptor protein, functions upstream of MALT1 and BCL10 in the NF-κB pathway. Mutations in CARD11, which enhance its ability to activate NF-κB signaling, can contribute to BCR pathway-independent survival and therapy resistance in CLL. Targeting MALT1 directly or disrupting the CARD11-MALT1 interaction holds the potential to overcome such resistance mechanisms by inhibiting this constitutive NF-κB activation. Dual inhibition of SHP-2 (a tyrosine phosphatase upstream of ERK) and MALT1 has shown synergistic suppression of CLL cell survival, particularly in venetoclax-resistant clones [121,122]. While still in preclinical development, such compounds may address resistance driven by microenvironmental or epigenetic adaptations. JNJ-67856633 is a selective MALT1 protease inhibitor being evaluated in early phase trials for B-cell malignancies. Though data in CLL remain preliminary, preclinical results indicate potent suppression of NF-κB signaling and synergy with BTK inhibitors [123].

FABP5 (fatty acid-binding protein 5) plays a key role in lipid metabolism and is involved in promoting tumor cell survival, immune evasion, and drug resistance by modulating metabolic and inflammatory signaling pathways [124]. Overexpression of FABP5 has been associated with poor prognosis in several cancers, making it a promising therapeutic target. AUR104 (VIJAY-1) is a FABP5 inhibitor, currently being investigated for the treatment of R/R CLL.

BMF-219 is a covalent menin inhibitor currently under investigation for the treatment of a range of hematologic malignancies, including CLL/SLL. Menin is a critical epigenetic regulator involved in gene transcription, particularly in cancers driven by KMT2A (also known as MLL1 rearrangements) and NPM1 mutations [125]. Inhibition of the menin–MLL1 interaction has shown promise in disrupting leukemogenic transcriptional programs, especially in acute leukemias, but its role is now being explored in other hematologic malignancies such as diffuse large B-cell lymphoma, multiple myeloma, and CLL [126].

c-MYC, a master transcription factor, is overexpressed in CLL cells with RT and in subsets with aggressive disease biology [127]. Its expression is stabilized through BCR and PI3K-AKT-mTOR signaling, making it a central node of interest. Omomyc, a c-MYC inhibitor acting via a dominant-negative mechanism, has shown preclinical efficacy in hematologic malignancies, although its development in CLL is nascent [128]. BRD4 inhibitors such as OTX015 (MK-8628) suppress MYC transcription and enhance venetoclax sensitivity [129]. Preclinical CLL models demonstrate synergy with BCL-2 and BTK inhibitors [130]. These agents may serve a niche role in high-risk or transformation-prone CLL.

Cyclin-dependent kinase 9 (CDK9) is a transcriptional kinase that phosphorylates RNA polymerase II, driving transcription of survival genes such as MCL-1 and MYC. CDK9 inhibition thus reduces anti-apoptotic protein levels and sensitizes cells to apoptosis [131]. Voruciclib, an oral CDK9 inhibitor, demonstrated preclinical synergy with venetoclax by downregulating MCL-1. AZD4573, a highly selective intravenous CDK9 inhibitor, was under investigation in a phase I/II trial (NCT03263637), with emerging data supporting its use in venetoclax-resistant CLL. Given that MCL-1 upregulation is a common mechanism of venetoclax resistance, CDK9 inhibition represents a rational strategy to restore sensitivity [132].

NEDD8-activating enzyme E1 (NAE1) inhibitor targets the c-Myc–Noxa axis [133]. By inhibiting NAE1, the neddylation of cullin-RING ligases is blocked, leading to the accumulation of proteins that promote apoptosis and cell cycle arrest, particularly effective in cancers driven by dysregulated c-Myc expression, such as CLL. Pevonedistat, an NAE1 inhibitor, is being investigated in a Phase I clinical trial (NCT03479268) in combination with ibrutinib for CLL [134].

Upregulation of the PI3K/AKT signaling pathway has emerged as a key mechanism driving resistance to BTK inhibitors in CLL. This pathway promotes cell survival, proliferation, and metabolic adaptation, providing leukemic cells with alternative signaling support when BTK is inhibited. Enhanced AKT activation has been documented in CLL patients who progress on noncovalent BTK inhibitors such as pirtobrutinib, indicating a compensatory mechanism that sustains malignant cell viability in the absence of effective BTK signaling [104]. In preclinical models, upregulation of the PI3K/AKT axis has been associated with continued NF-κB activation and metabolic adaptation following BTK inhibitor therapy, offering an explanation for relapse in patients lacking classical resistance mutations [135]. Inhibiting these downstream or parallel survival pathways has therefore emerged as a rational strategy to restore treatment sensitivity. Given this, the use of AKT inhibitors presents a promising therapeutic strategy. Two such agents, MK2206 and AZD5363 (capivasertib), have been investigated for their antitumor activity across various malignancies, including B-cell lymphomas. MK2206 is an allosteric inhibitor of AKT that has shown preclinical synergy with both ibrutinib and venetoclax, potentially enhancing apoptosis in resistant CLL cells [136]. Similarly, AZD5363, a pan-AKT inhibitor, has demonstrated the ability to suppress AKT-mediated signaling in hematologic malignancies and is under investigation in clinical trials [137]. These agents may be particularly effective when used in combination with BTK inhibitors to suppress compensatory signaling and improve treatment durability.

To simultaneously block multiple survival pathways, triple kinase inhibitors targeting PI3K/AKT/mTOR or BTK/SYK/PI3K have been developed [138]. These agents aim to reduce pathway redundancy and delay resistance emergence. SF1126, a pan-PI3K and BRD4 inhibitor, demonstrated preclinical synergy with ibrutinib and venetoclax in resistant models for B-cell non-Hodgkin’s lymphoma [139]. Q702 is a triple kinase inhibitor targeting AXL, Mer, and CSF1R, currently being investigated [140]. AXL and Mer are members of the TAM receptor family and are often overexpressed in CLL. CSF1R is critical for the survival and function of tumor-associated macrophages, which can promote a pro-tumorigenic microenvironment.

Keynatinib is a third-generation, orally available inhibitor of the epidermal growth factor receptor (EGFR) mutant form T790M, with potential antineoplastic activity [141]. Upon administration, keynatinib binds to and inhibits EGFR T790M, a secondarily acquired resistance mutation, inhibits the tyrosine kinase activity of EGFR T790M, prevents EGFR T790M-mediated signaling, and leads to cell death in EGFR T790M-expressing tumor cells. EGFR, a receptor tyrosine kinase that is mutated in many tumor cell types, plays a key role in tumor cell proliferation and tumor vascularization.

## 7. Conclusions and Future Perspectives

The emergence of resistance to both BTK and BCL-2 inhibitors marks a critical turning point in the treatment of CLL, particularly for patients with double-refractory disease. The expansion of targeted therapies, including novel cellular therapies, T-cell engagers, engineered antibodies, and small molecule inhibitors, represents a dynamic and rapidly evolving therapeutic frontier. These approaches offer the potential to overcome existing resistance mechanisms and provide durable responses in a patient population with limited options.

From a clinical perspective, therapies such as BTK degraders are particularly promising given their ability to bypass established resistance mechanisms at the molecular level. CAR T-cell therapy, while limited by access and immune dysfunction in some patients, has demonstrated deep and durable responses, especially when used in combination with BTK inhibitors. Additionally, bispecific and trispecific T-cell engagers are emerging as viable off-the-shelf immunotherapeutic options with growing evidence of activity in heavily pretreated CLL. Other novel immunotherapeutic strategies continue to expand the frontier of T-cell-based approaches in CLL. While BCMA is expressed at low levels in CLL, preclinical studies have shown that the BCMA × CD3 bispecific antibody teclistamab can activate both healthy donor and autologous CLL-derived T cells, leading to targeted cytotoxicity even without γ-secretase enhancement [142]. Other strategies designed to restore or augment cytotoxic T-cell function include the use of Vγ9Vδ2-T cells, which possess innate antitumor activity but are often dysfunctional in CLL [143]. Ex vivo stimulation and ibrutinib-induced phenotypic modulation may restore their function. Furthermore, Siglec-6, a surface protein enriched on CLL cells, has recently emerged as a promising target for bispecific antibodies that redirect T-cells, showing selective in vitro and in vivo cytotoxicity [144]. These emerging therapies could complement existing immunotherapy platforms and further personalize treatment approaches.

Despite this progress, several challenges remain. The optimal sequencing of these therapies is undefined, and long-term outcomes with many agents are still immature. Moreover, better understanding of tumor microenvironmental resistance, immune exhaustion, and antigen escape will be essential to maximize efficacy. Future research must focus on integrating biomarker-driven strategies, improving MRD-guided endpoints, and expanding access to advanced therapies through simplified manufacturing platforms and in vivo approaches.

MRD has also emerged as a powerful biomarker for assessing treatment depth and predicting long-term outcomes in CLL. MRD-guided therapeutic approaches, such as those being studied in the FLAIR trial (NCT03462719), provide a proof of principle for tailoring therapy duration and intensity based on individual response dynamics. Incorporating MRD assessment into routine clinical decision-making may optimize outcomes while minimizing toxicity.

Another urgent clinical challenge is Richter transformation (RT), an aggressive evolution of CLL that remains associated with poor prognosis and limited therapeutic options. RT is driven by distinct molecular events such as TP53 disruption, MYC overexpression, and NOTCH1 mutations. These biological features present potential therapeutic targets, and novel strategies, including immune checkpoint inhibitors, EZH2 inhibitors, and therapies modulating MYC and PI3K pathways, are actively under investigation. A recent review by Maher et al. highlights the complex biology of RT and the numerous avenues for targeted intervention [145]. Addressing RT with biologically informed, mechanism-based approaches represents a critical priority for future clinical research.

Continued use of genomic profiling and resistance mutation analysis will be essential in guiding the clinical use of these agents. Moreover, rational combinations and adaptive trial designs will be key to overcoming resistance and improving survival outcomes for patients with advanced or double-refractory CLL. As clinical trials continue to investigate these agents, there is hope that one or more of these novel approaches will ultimately revolutionize the therapeutic landscape for this difficult-to-treat patient group.

Finally, while these innovations offer hope, they are accompanied by significant costs. Financial toxicity associated with novel targeted therapies and cellular treatments remains a major barrier to access, especially in real-world settings. Future efforts must therefore also focus on improving affordability, reimbursement, and infrastructure to ensure equitable access to these potentially life-saving treatments.

With continued translational research and patient-focused trial design, there is real potential for these innovations to transform care in CLL, moving us closer to the goal of achieving sustained remissions, even in the most treatment-refractory settings.

## Figures and Tables

**Table 1:** Active clinical trials of various cellular therapies in CLL.

Agent Name	Target	Study Design/Phase	Clinicaltrial.gov Identifier	Population, Sample Size (Estimated)
Single target CAR T-cell				
MB-106	CD20	I/II	NCT03277729	53, R/R B Cell-NHL, R/R CLL, Other
huCART19-IL18	huCART19-IL18	I	NCT04684563	72, CLL, NHL, ALL
CAR-37 T Cells	CD37	I	NCT04136275	6, CD37+ hematologic malignancies
BAFFR-targeting CAR T Cells	BAFFR	I	NCT05370430	36, R/R B-NHL
BAFFR-targeting CAR T Cells	BAFFR	I	NCT06191887	26, R/R BAFFR-Expressing B-Cell Hematologic Malignancies
CARCIK-CD19	CD19 (Allogenic)	I/II	NCT05869279	29, B-cell NHL, CLL
ALLO-501A, ALLO-647, ALPHA2 study	CD19 (Allogenic)	I/II	NCT04416984	160, Large B Cell Lymphoma, CLL, SLL
SC291-101, ARDENT study	CD19 (Allogenic)	I	NCT05878184	16, NHL, CLL
UB-VV111	CD19 (in vivo)	I	NCT06528301	106, R/R LBCL, CLL
FT819	CD19	I	NCT04629729	54, R/R B-cell Lymphoma, CLL and Precursor B-cell ALL
Double target CAR T-cell				
ATLCAR.κ.28	Kappa-CD28	I	NCT04223765	20, R/R κ+ mantle cell and indolent NHL
CD19/CD22 Bicistronic CAR	CD19/CD22	I/II	NCT05442515	116, ALL or related B-cell lymphoma
CD19/CD22 CAR	CD19/CD22	I/II	NCT03614858	20, R/R B-Cell ALL.
CD19/CD22 CA	CD19/CD22	I	NCT03448393	44, CD19+CD22+ B cell ALL, isolated CNS ALL, or lymphoma
CAR2219 CAR	CD19/CD22	I/II	NCT06834529	20, R/R CD19/CD22 positive B cell Leukemia and Lymphoma
IL-7/IL-15 manufactured CD20/19 CAR	CD-20/19	I/II	NCT04186520	100, R/R B Cell Malignancies
CD19-BAFF CAR	CD19-BAFF	I	NCT06346912	20, data B-cell ALL and B-cell NHL
Triple target CAR T-cell				
CD19/CD20/CD22 CAR	CD19/CD20/CD22	I	NCT05418088	54, R/R NHL, ALL, CLL, B-PLL
Other Cellular Therapies				
IOV-2001 (Adoptive Cell Therapy)	autologous PBL	I/II	NCT04155710	7, R/R CLL/SLL
CHARKALL (Adoptive Transfer of Autologous T Lymphocytes)	Kappa-CD28 T-cells	I	NCT00881920	54, CLL, B cell NHL, MM who express Kappa-light chain
ATECRAB	autologous or syngeneic PBTLs and EBV-CTLs expressing CD19	I	NCT00709033	3, B cell NHL, CLL
ANCHOR2	KUR-502, Allogeneic NK T-Cells Expressing CD19	I	NCT05487651	36, R/R B-cell NHL, ALL, CLL
NKX019	NK (Intravenous allogenic)	I	NCT05020678	150, B-cell Malignancies
ANCHOR	Allogenic CD19- NK	I	NCT03774654	48, R/R B-cell Malignancies
Allogenic CD19-CAR-NK	Allogenic CD19- NK	I	NCT05739227	12, R/R B-cell Malignancies
NK cells combination with IL-2 and vactosertib	IL-2 and TGFβ receptor 1 NK	I	NCT05400122	12, Colorectal Cancer, Gastric/esophageal Cancer, and R/R Hematologic Malignancies

NK = natural killer, NHL = non-Hodgkin lymphoma, ALL = acute lymphoblastic leukemia, LBCL = large B-cell lymphoma, MM = multiple myeloma, PLL = prolymphocytic leukemia, PBL = peripheral blood lymphocytes, PBTLs = peripheral blood T lymphocytes, EBV-CTLs = Epstein–Barr virus-specific cytotoxic T lymphocytes.

**Table 2:** Active clinical trials evaluating T-cell engagers in CLL.

Agent Name	Engaging Targets	Study Design/Phase	Clinicaltrial.gov Identifier	Population, Sample Size
Bispecific T-cell Engagers				
NVG-111	ROR1 ×CD3	I	NCT04763083	90, R/R ROR1+ Malignancies- CLL, SLL, MCL, FL, DLBCL, NSCLC, malignant melanoma
ONO-4685	PD-1 × CD3	I	NCT06547528	108 T cell Lymphoma, CLL/SLL
JNJ-75348780	CD3 × CD22	I	NCT04540796	147, R/R B-cell NHL, CLL
MGD024	CD123 × CD3	I	NCT05362773	130, R/R hematological malignancies
Trispecific T-cell engagers				
TITANium, AZD5492	CD8/TCR × CD20	I/II	NCT06542250	174, R/R B-Cell Malignancies
CC312	CD19 × CD3 × CD28	I	NCT06037018	44, R/R CD19 Positive B-cell Hematologic Malignancies
Tetraspecific T-cell Engagers				
GNC-035	CD3 × 41BB × PD-L1 × ROR	I/II	NCT05944978	40, R/R CLL, other hematological malignancies

ROR1 = receptor tyrosine kinase-like orphan receptor 1, MCL = mantle cell lymphoma, DLBCL = diffuse large B-cell lymphoma, FL = follicular lymphoma, NSCLC = non-small cell lung cancer, NHL = non-Hodgkin lymphoma.

**Table 3:** Active clinical trials of monoclonal antibodies and antibody–drug conjugates in CLL.

Agent Name	Targets	Study Design/Phase	Clinicaltrial.gov Identifier	Population, Sample Size
Belimumab (BeliVeR)	BAFF	II	NCT05069051	120, R/R CLL
MOR00208	CD19	II	NCT02005289	41, R/R or Old untreated CLL, SLL or PLL
CAP-100	Humanized C-C-chemokine Receptor 7	I	NCT04704323	18, R/R CLL
Aplitabart (IGM-8444)	IgM DR5	I	NCT04553692	272, R/R solid or hematologic cancers
Zilovertamab Vedotin (MK-2140), (waveLINE-006)	Antibody-drug conjugate: ROR1 and monomethyl auristatin E	II	NCT05458297	223, MCL, RTL, FL, CLL

indolent chronic lymphocytic leukemia (CLL)/small lymphocytic lymphoma (SLL), mantle cell lymphoma (MCL), mantle cell lymphoma (MCL), Richter’s transformation lymphoma (RTL), follicular lymphoma (FL), and chronic lymphocytic leukemia (CLL).

**Table 4:** Small molecule inhibitors far advanced in clinical development in CLL.

Agent Name	Targets	Study Design/Phase	Clinicaltrial.gov Identifier	Population, Sample Size	Efficacy (ORR, PFS, OS, Median Follow-Up
Sonrotoclax	BCL-2 inhibitor	III	NCT04277637	46 RR CLL/SLL	Median follow up 19.3 months Sontroclax + zanabrutinib ORR 97% (CR 57%) for all doses, 100% ORR (CR 73%) for 320 mg dose [93]
		I/II	NCT04277637	112 TN CLL/SLL	Median follow up 18.3 months ORR 100% Best uMRD 90% [94]
Lisaftoclax (APG-2575)	BCL-2 inhibitor	I	NCT04215809	176, 22 TN and 154 R/R CLL/SLL	ORR- lisaftoclax plus acalabrutinib—96.6% ORR—85.7%, 100%, and 66.7% in the ven-exposed, ven-exposed but BTKi-naïve, and ven- and BTKi-exposed pts, respectively [95].
Nemtabrutinib	BTK (non-covalent)	I/II	NCT03162536	112	ORR 56% (42–69) mPFS 26.3 months ORR in double exposed 58% (37–78) mPFS in double exposed 10.1 months [96]
Orelabrutinib	Irreversible BTK	II	NCT03493217	80, R/R CLL/SLL	ORR 93.8% (86.01–97.94) CR 23.8% [97]
LP-168	Dual covalent + non-covalent BTK	I	NCT04775745	R/R B-cell malignancies	ORR 54.5% Median follow up 12.6 months In Gatekeeper mutation CLL patients ORR 77.8%, median follow up 14 months [98,99]
NX-5948	BTK Degrader	I	NCT05131022	87, B-cell malignancies, 34 CLL	ORR 76.7% [100]
BGB-16673	BTK Degrader	I/II	NCT05006716	49, R/R CLL, WM, MCL, MZL, DLBCL, FL, or RT	ORR 77.6% [101]
NX-2127	BTK + Ikaros/Aiolos degrader	I	NCT04830137	47, R/R B-cell malignancies	Median follow up 9.5 months [102]. ORR- not reported

overall response rate (ORR), progression-free survival (PFS), overall survival (OS), undetectable minimal residual disease (uMRD), treatment-naïve (TN) R/R CLL, Waldenstrom Macroglobulinemia (WM), marginal zone lymphoma (MZL), mantle cell lymphoma (MCL), non-germinal center B-cell diffuse large B-cell lymphoma (DLBCL), follicular lymphoma (FL), or Richter transformation (RT).

**Table 5:** Small molecular inhibitors in early phases of clinical trials in R/R CLL.

Agent Name	Targets	Study Design/Phase	Clinicaltrial.gov Identifier	Population, Sample Size
ACY-1215 (Ricolinostat)	Histone deacetylase inhibitor, HDAC6	I	NCT02787369	3, R/R CLL
AZD6738 (Ceralasertib)	ATR kinase	I	NCT03328273	11, R/R High-risk CLL
DZD8586 (TAI-SHAN8)	LYN/BTK	II	NCT06539182	155, R/R CLL
ONO-7018	MALT1	I	NCT05515406	108, R/R NHL or CLL
JNJ-67856633	MALT1	I	NCT04876092	45, B-cell NHL, CLL
JNJ-67856633	MALT1	I	NCT03900598	266, R/R B-cell NHL, CLL
AUR104 (VIJAY-1)	FABP5	I	NCT06761586	42, R/R NHL or CLL
BMF-219	Covalent Menin	I	NCT05153330	55, AML, ALL (With KMT2A/ MLL1r, NPM1 Mutations), DLBCL, MM, and CLL/SLL
IDP-121 (CASSANDRA)	c-MYC	I/II	NCT05908409	37, MM, DLBCL-NOS, HGBL-DH/TH, HGBL-NOS, CLL
MLN4924, TAK924 (Pevonedistat)	Nedd8-activating enzyme E1 regulatory subunit (NAE1)	I	NCT03479268	18, R/R CLL, NHL
SLS009	CDK9	I/II	NCT04588922	160, R/R AML, lymphoma/CLL/SLL
Voruciclib	CDK9	I	NCT03547115	100, R/R B-Cell Malignancies or AML
PRT2527	CDK9	I	NCT05665530	86, R/R Hematologic Malignancies
Keynatinib (TL007)	EGFR	I	NCT04807881	75, R/R-PCNSL, CLL/SLL, MCL
Q702	Axl/Mer/CSF1R Triple Kinase	I	NCT06712810	Estimated enrollment 46, Hematological malignancies

Fatty acid-binding protein 5 (FABP5), multiple myeloma (MM), diffuse large B cell lymphoma not otherwise specified (DLBCL-NOS), high-grade B cell lymphoma with double or triple hit rearrangement (HGBL-DH/TH) and HGBL-NOS, and chronic lymphocytic leukemia (CLL).
